# Supplementary material for: Removal of Total Nitrogen and Phosphorus Using Single or Combinations of Aquatic Plants
Source: Int J Environ Res Public Health. 2019 Nov 22;16(23):4663. doi: 10.3390/ijerph16234663 (PMC6926882; doi:10.3390/ijerph16234663)
Supplement: Supplementary file 1 [file ijerph-16-04663-s001.pdf]

Supplementary data

Removal of total nitrogen and phosphorus using single or combinations of aquatic plants

Feng Su [1,4], Zhian Li [1,3], Yingwen Li [1], Lei Xu [1,4], Shiyu Li [1,4], Yongxing Li [1], Hongfeng Chen [1,2], Ping Zhuang [1,3,*], and Faguo Wang [1,2,*]

South China Botanical Garden, Chinese Academy of Sciences, Guangzhou 510650, PR China

[2] Key Laboratory of Plant Resources Conservation and Sustainable Utilization, Guangdong Provincial Key Laboratory of Applied Botany, South China Botanical Garden, Chinese Academy of Sciences, Guangzhou 510650, PR China

[3] Southern Marine Science and Engineering Guangdong Laboratory (Guangzhou), Guangzhou 511458, PR China

[4] University of Chinese Academy of Sciences, Beijing 100049, PR China

* Correspondence: zhuangp@scbg.ac.cn (Z.P.), Tel.: +86-20-37252905, Fax: +86-20-37252905; wangfg@scbg.ac.cn (F.W.)

Table S1. Biomass production of aquatic plants in the screening experiments grow for 36 days (n=3)

	Initial biomass (day 1)	Final biomass (day 36)	Mean net increase (g/tank)	Relative biomass increase
Colocasia tonoimo	203±10.34	163±10.05	-42.08	-20.4%
Dysophylla sampsonii	102±0.14	32.0±4.38	-69.51	-68.5%
Eichhornia crassipes	177±6.89	230±37.33	52.74	46.0%
Eleocharis plantagineiformis	259±68.00	230±2.67	-29.00	-13.0%
Hydrocotyle vulgaris	238±27.17	385±20.13	146.7	64.1%
Ipomoea aquatica	150±0.36	156±4.24	5.73	3.8%
Rotala indica	149±0.93	163±2.12	13.82	9.2%
Salvinia natans	100±0.06	214±7.18	113.87	114.0%
Typha orientalis	466±0.29	377±15.68	-88.50	-19.0%

Table S2. Plant biomass development during treatment (g/tank, n=3, fresh weight)

Plants	Artificial domestic wastewater						Artificial animal farm wastewater					
	Initial	Day 11	Day 36	Day 48	Total	Increase	Initial	Day 11	Day 36	Day 48	Total	Increase
E. plantagineiformis	74.8	11.7	0.00	94.0	106	41.4%	67.2	10.2	0.00	52.4	62.5	-6.91%
Hydrocotyle vulgaris	67.1	4.7	5.50	46.6	56.8	-15.3%	71.1	3.21	30.5	26.2	59.9	-15.7%
Ipomoea aquatica	46.3	36.2	25.4	22.0	83.5	80.5%	48.1	22.4	7.96	21.8	52.1	8.44%
Salvinia natans	39.8	66.1	45.5	22.1	133.7	235%	39.7	27.6	36.2	25.5	89.3	125%
Salvinia natans + *E. plantagineiformis*	107	78.8	38.0	56.6	173	62.8%	120	9.68	13.6	92.0	115	-4.20%
Hydrocotyle vulgaris +*E.plantagineiformis*	147	13.9	0.00	79.9	93.7	-36.3%	149	18.1	0.00	86.3	104	-25.1%
Ipomoea aquatica +*E.plantagineiformis*	127	47.4	15.2	71.2	134	5.14%	128	20.8	14.7	13.9	149	16.4%
Salvinia natans +*E.plantagineiformis* + *H.vulgaris*	87.8	24.1	19.0	75.4	118	34.9%	87.4	8.33	18.9	97.8	125	27.9%

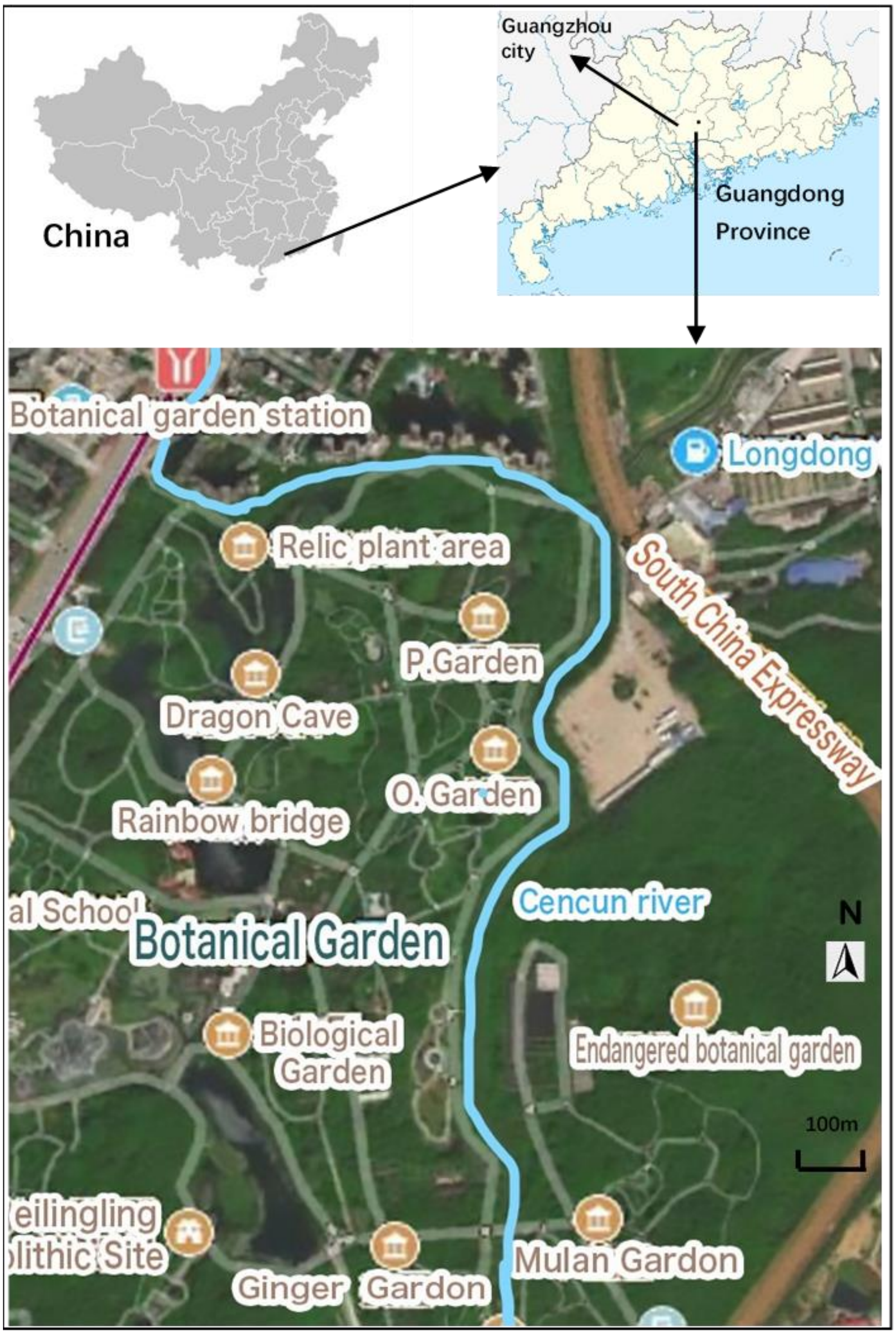

Figure 1. Locations of the drainage ditch (Cencun River) flowing through the South China Botanical Garden, Guangzhou, China.

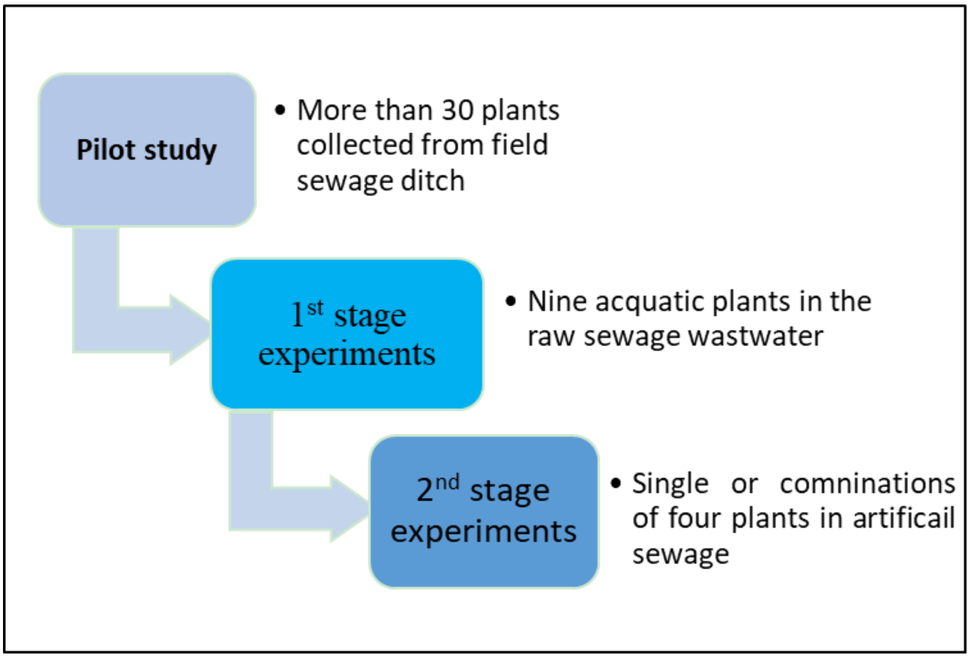

Figure S2. The flowchart of this study.
